# BLV-CoCoMo-qPCR: estimation of bovine leukemia virus (BLV) proviral load harbored by lymphocyte subpopulations in BLV-infected cattle at the subclinical stage of enzootic bovine leucosis

**DOI:** 10.1186/1742-4690-11-S1-P143

**Published:** 2014-01-07

**Authors:** Yoko Aida, Shin-nosuke Takeshima, Carlos Javier Panei, Takashi Omori, Tetsuo Nunoya, William C Davis, Hiroshi Ishizaki, Kazuhiro Matoba

**Affiliations:** 1 Viral Infectious Diseases Unit, RIKEN, 2-1 Hirosawa, Wako, Saitama, Japan; 2 CONICET and Virology Laboratory, Faculty of Veterinary Sciences, National University of La Plata, Argentina; 3 Nippon Institute for Biological Science, Tokyo, Japan; 4 Department of Veterinary Microbiology and Pathology, Washington State University, Pullman, WA, USA; 5 NARO Institute of Livestock and Grassland Sciences, Tochigi, Japan

## 

Bovine leukemia virus (BLV) is associated with enzootic bovine leukosis (EBL), which is the most common neoplastic disease of cattle. The present study reports the distribution of BLV provirus in peripheral blood mononuclear cell subpopulations isolated from BLV-infected cows at the subclinical stage of EBL as examined by cell sorting and a new quantitative real-time PCR method, BLV-CoCoMo-qPCR, to measure the proviral load of both known and novel BLV variants in BLV-infected animals. Phenotypic characterization of five BLV-infected but clinically normal cattle with a proviral load of > 100 copies per 1 x 10^5^ cells identified a high percentage of CD5^+^ IgM^+^ cells (but not CD5^-^IgM^+^ B cells, CD4^+^ T cells, or CD8^+^T cells). These lymphocyte subpopulations were purified from three cattle by cell sorting or using magnetic beads, and the BLV proviral load was estimated using BLV-CoCoMo-qPCR. The CD5^+^ IgM^+^ B cell population in all animals harbored a higher BLV proviral load than the other cell populations. The copy number of proviruses infecting CD5^-^ IgM^+^ B cells, CD4^+^ cells, and CD8^+^ T cells (per 1 ml of blood) was 1/34 to 1/4, 1/22 to 1/3, and 1/31 to 1/3, respectively, compared with that in CD5^+^ IgM^+^ B cells. Moreover, the BLV provirus remained integrated into the genomic DNA of CD5^+^ IgM^+^ B cells, CD5^-^ IgM^+^ B cells, CD4^+^ T cells, and CD8^+^ T cells, even in BLV-infected cattle with a proviral load of <100 copies per 10^5^ cells.

